# Australian Dentists' Knowledge of the Consequences of Interpretive Errors in Dental Radiographs and Potential Mitigation Measures

**DOI:** 10.1002/cre2.70027

**Published:** 2024-10-17

**Authors:** Shwetha Hegde, Shanika Nanayakkara, Stephen Cox, Rajesh Vasa, Jinlong Gao

**Affiliations:** ^1^ Dentomaxillofacial Radiology Sydney Dental School, University of Sydney Sydney Australia; ^2^ Sydney Dental School Institute of Dental Research, Westmead Centre for Oral Health, University of Sydney Sydney Australia; ^3^ Discipline of Oral Surgery Sydney Dental School, University of Sydney Sydney Australia; ^4^ Translational Research and Development, Applied Artificial Intelligence Deakin University Melbourne Australia

**Keywords:** dental radiography, medical errors, patient safety, radiographic image interpretation

## Abstract

**Objectives:**

Dental radiographs, typically taken and interpreted by dentists, are essential for diagnosis and effective treatment planning. Interpretive errors in dental radiographs, stemming from failures of visual and cognitive processes, can affect both patients and clinicians. This survey aimed to assess the dental practitioners' perceptions of the consequences of these errors and potential measures to minimize them.

**Materials and Methods:**

This online anonymized survey assessed Australian dental practitioners' perceptions of the consequences of these errors and potential mitigation measures using ranking, Likert scale, and open‐ended questions. The data were analyzed using descriptive statistics and bivariate analysis.

**Results:**

Participants identified undertreatment (72%) and legal implications (82%) as the most significant consequences of interpretive errors, whereas severe harm to patients was deemed the least likely. Dental practitioners placed a greater emphasis on maintaining a high level of competence and the well‐being of their patients. Utilizing high‐quality images (63.9%) and appropriate radiographs (59.7%) were identified as the most effective measures to minimize interpretive errors. Participants showed hesitancy regarding the reliance on machine learning as a clinical decision‐making tool.

**Conclusions:**

The survey provides valuable practical insights into the consequences and targeted measures to minimize the occurrence of interpretive errors. Efforts to minimize interpretive errors should address patient safety and practitioners' concerns about professional reputation and business viability. The study also suggests further research into the role of machine learning algorithms in reducing interpretive errors in dentistry.

## 1 Introduction

A great deal of attention and effort is dedicated to ensuring the provision of safe and high‐quality care to patients. Nevertheless, mistakes arise from radiographic interpretive errors, leading to unintentional patient harm caused by medical management rather than the patient's underlying condition (Kohn, Corrigan, and Donaldson 2000). Interpretive errors are a type of diagnostic error that has been extensively studied in medical radiology (Degnan et al. 2019; Itri et al. 2018; Waite et al. 2017). Although interpretive errors in dental radiographs have not been as widely studied as in medical radiology, evidence suggests that they occur in dental practice, and several factors influence their occurrence (Hegde et al. 2024). These factors include time pressure, stress, clinical experience, case complexity, and cognitive load (Bretherton, Chapman, and Chipchase 2016; Cave and Hutchison 2019; Hegde et al. 2022; Plessas et al. 2018, 2019).

The severity of consequences arising from interpretive errors varies and depends on factors such as the type of error, the stage of the diagnostic process, and the promptness of identification and correction (Balogh et al. 2015). These errors can lead to adverse outcomes that can be classified as patient‐related or clinician‐related consequences, potentially resulting in unnecessary treatments, malpractice lawsuits, damage to the clinician's reputation and credibility, increased financial burdens for both the clinician and patient, and a negative impact on the patient's quality of life (Nikdel et al. 2018). Among the consequences of interpretive errors in medical imaging, malpractice lawsuits have been particularly significant (Busardò et al. 2015). Although extreme consequences of interpretive errors in dentistry are unlikely, it is essential to acknowledge and mitigate these errors to ensure quality patient care and minimize associated consequences. Furthermore, the context of dental imaging, radiographic interpretation, and diagnosis is distinct in dentistry compared to medicine. General dentists are responsible for capturing and interpreting dental plain film radiographs, whereas in medicine, specialists perform most imaging and interpretation of medical radiographs. This distinction could lead to consequences of interpretive errors that are unique to dentistry. Given their role in initial radiographic screening, general dentists play a crucial role in the early detection and diagnosis of dental conditions (Rushton, Horner, and Worthington 2001; Zeichner, Ruttimann, and Webber 1987).

With advances in diagnostics and healthcare technologies, healthcare is poised to minimize errors and improve diagnostic accuracy and quality of patient care. However, there is no single solution to the problem of interpretive errors. In medical imaging, several approaches have been applied to improve accuracy in radiographic diagnosis. These include an emphasis on critical analysis and clinical decision‐making in medical education (Morrissey and Heilbrun 2017), the use of simulation in teaching diagnostic methods (Solomon et al. 2021), diagnostic checklists for structured analysis of medical images (Ely, Graber, and Croskerry 2011), and improvements in technology such as automated clinical decision support (Oakden‐Rayner 2019) and machine learning (ML) algorithms (Choy et al. 2018). Using technology can be advantageous in minimizing interpretive errors as it can enhance human performance. Peer review with continuous and critical reflection of physicians' performance using structured procedures has also been shown to improve diagnostic accuracy (Lee et al. 2013). Active and effective management of interpretive errors requires the development of a system for identifying and managing these errors. Awareness and knowledge of the frequency and the causes are prerequisites for identifying interpretive errors as and when they occur in clinical practice. These steps will enable the implementation of effective measures to prevent such errors from occurring in the future.

This survey aims to gather dental practitioners' views on the impact of interpretive errors on both themselves and their patients. This study also aims to assess dental practitioners' perceptions of the currently available measures to mitigate such errors in clinical practice.

## 2 Materials and Methods

A cross‐sectional, anonymized survey of dentists practising in New South Wales, Australia, was undertaken. This survey was conducted as a part of a broader study that assessed the perceptions of Australian dental practitioners about interpretive errors in dentistry (Hegde et al. 2024). This study was approved by the University of Sydney Human Research Ethics Committee (Approval Number 2020/336) and conducted in accordance with the Declaration of Helsinki (World Medical Association 2013). The target population included dental practitioners within the research team's network, subscribers to the ADA's e‐newsletters, users of social media platforms, and attendees of dental continuing professional development (CPD) workshops. An anonymized link to the survey was created and made available to potential participants via emails, e‐newsletters, social media platforms, and during CPD workshops. Informed consent was assumed when the participants completed and submitted the survey.

The survey was conducted in two phases: the first from September 2020 to July 2021, and the second from January to March 2022, with the latter aimed at increasing participant numbers. Regular monitoring of the data during these periods allowed for the observation of response trends. Data saturation was determined manually, evidenced by the lack of new information arising from subsequent responses, a method analogous to the data saturation techniques employed in qualitative studies. The survey concluded once it was established that data saturation had been achieved, in line with the principles of grounded theory (Glaser and Strauss 2017). The online questionnaire was developed and distributed on the Qualtrics platform (www.qualtrics.com). After conducting an extensive review of literature pertaining to interpretive errors in radiology and clinical decision‐making, as well as consulting experts in radiology and survey design, a questionnaire was created. This questionnaire aimed to explore how participants perceived the consequences of interpretive errors in dental radiographs and to explore their views on measures to minimize interpretive errors in clinical dentistry. The questionnaire comprised several categories, including questions about the consequences of interpretive errors for both clinicians and patients and measures to reduce such errors in dentistry. Demographic data gathered included participants' location and type of practice, the type and the number of radiographs taken in their dental practice each day, and the type of intra‐oral imaging system used in their practice. The survey questionnaire is included in Supporting Information S1: File 1, with questions in Sections 5, 6, and 7 addressing this study. These sections included questions about the consequences of interpretive errors, potential solutions to minimize errors, and participant demographic data (Supporting Information Material).

Data were exported from the Qualtrics platform to SPSS statistical software (Version 26, IBM, SPSS, Chicago, IL) for analysis. Cronbach's *⍺* was used to measure the internal consistency of the survey instrument. Descriptive statistics were used to summarize the demographic data of participants. Chi‐squared tests of independence were used to find the association between categorical variables. Fisher's test was used when more than 20% of the cells had an expected frequency of less than 5%. Statistical comparisons were not conducted when expected cell counts were less than 1. The responses between different demographic groups, such as gender, type of practice (public or private), and level of training (general dentist or specialist), were compared. Spearman's rank correlation was used to test the correlations between variables. The statistical significance was set at *p* < 0.05.

## 3 Results

This survey received 118 responses, of which 38 records were excluded from the analysis due to missing data. The survey instrument had a high internal consistency with Cronbach's *⍺* value of 0.928. The significant findings from the survey are presented here to highlight meaningful trends and insights.

### 3.1 Demographic Features

A summary of the demographic characteristics of the survey participants is presented in Table 1. The participants' ages ranged from 23 to 72 years. The reported clinical experience ranged from 1 to 50 years, averaging 17.1 (±11.8) years. On average, the participants reported taking 12 radiographs a day: four periapical radiographs, five bitewings, and three OPGs. The participants reported taking a range of radiographs, and most of them used the photostimulable phosphor (PSP) imaging system.

**Table 1:** Demographic characteristics of the participants.

Characteristics	Value (*n* = 80)
Age (years)	
Mean (SD)	41.7 ± 11.0
Minimum	23
Maximum	72
Gender, *n* (%)	
Female	46 (57.5)
Male	22 (27.5)
Not specified	12 (15.0)
Clinical experience in years	
Mean (SD)	17.1 (±11.8)
Minimum	1
Maximum	50
Type of clinical practice (choice count) *n* (%)	
Type of practice^a^	
General	35 (43.7)
Specialist	14 (17.5)
Not specified	31 (38.8)
Sector^b^	
Private	21 (26.3)
Public	14 (17.5)
Both	11 (13.8)
Practice location^b^	
Metropolitan	13 (16.4)
Regional	5 (6.3)
Other (university academic)	2 (0.03)
Time spent doing different activities per week (mean hours [SD])	
Clinical	27.90 (±12.5)
Research	3.09 (±5.4)
Teaching	5.76 (±7.5)
Administration	4.83 (±5.9)
Patients per day requiring radiographs (mean ± SD)	6.2 (±2.5)
Number of radiographs taken per day (mean ± SD)	
Periapical	3.8 (±3.4)
Bitewings	4.9 (±3.7)
Panoramic radiographs (OPG)	2.7 (±2.7)
Other	0.49 (±1.0)
Imaging systems used in clinical practice, *n* (%) (choice count)^b^	
Photostimulable phosphor (PSP)	42 (52.5)
Direct digital (CCD, CMOS)	25 (31)
Analog films (chemically processed)	14 (17.5)
Other	20 (25)

^a^ The total number of responses does not add up to 100% for some variables due to missing responses.

^b^ As the participants have selected more than one choice, the responses don't add up to 100%.

### 3.2 Consequences of Interpretive Errors Affecting Patients and Clinicians

**Figure 1:**
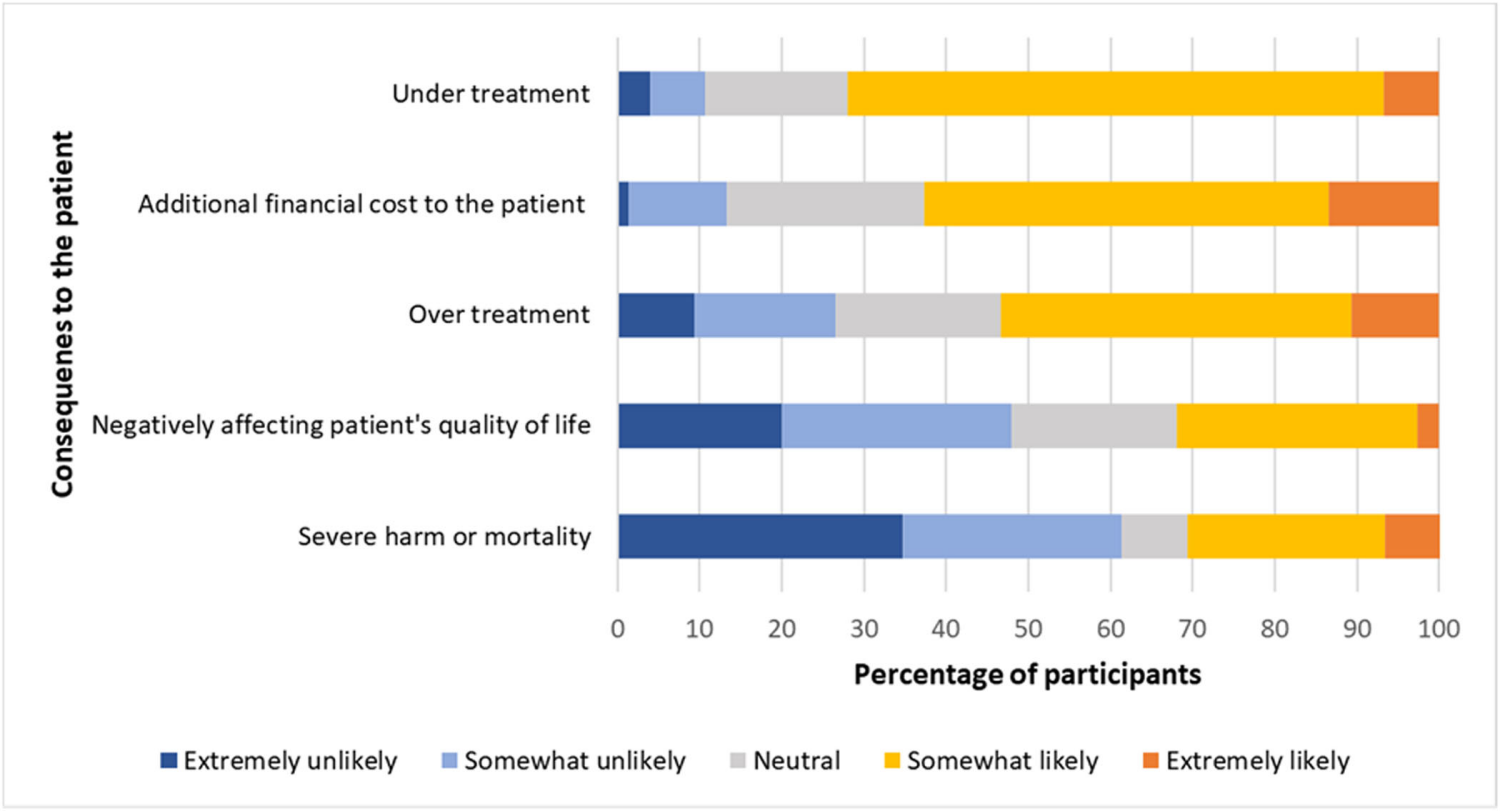
Likelihood of consequences of interpretive errors affecting the patient.

**Figure 2:**
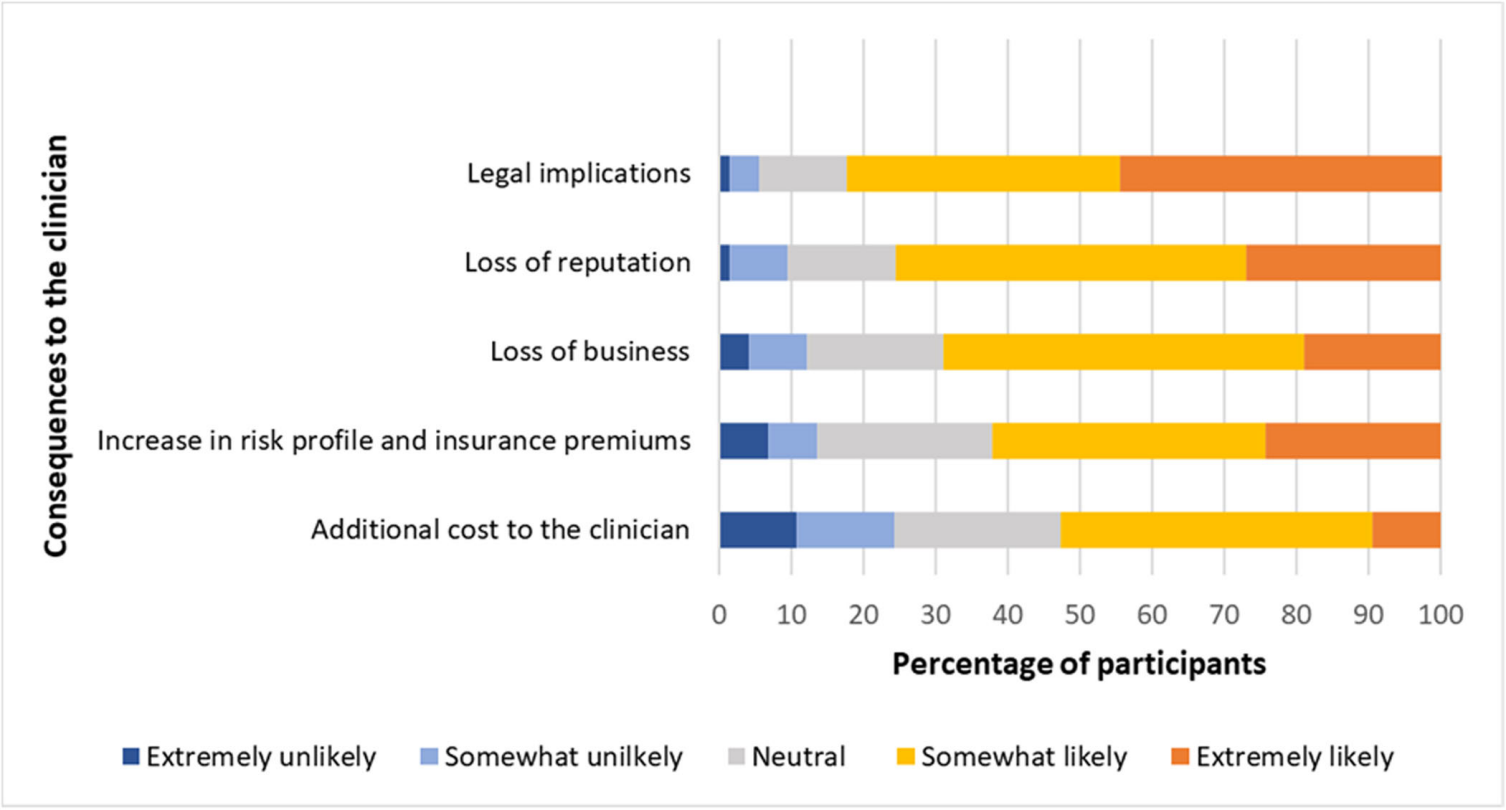
Likelihood of consequences of interpretive errors affecting the clinician.

Among the patient‐related consequences, the participants recognized undertreatment (72%) as the most significant, followed by an increase in the financial burden to the patient (62%). In contrast, severe harm or patient mortality (30.7%) was considered the least likely outcome (Figure 1). For clinician‐related consequences, participants identified legal consequences (82%) as the most likely ones. Loss of reputation (75.6%) was also recognized as a significant consequence, whereas the additional cost to the clinician (52.7%) was considered as the least likely consequence (Figure 2).

Statistically significant correlations were observed between patient‐related and clinician‐related consequences (Table S1). Additional cost to the dentist was the only one that correlated to all patient‐related and clinician‐related consequences with statistical significance (*p* < 0.001). Similarly, additional cost to the patient had a moderate correlation to all other patient‐related consequences (*p* < 0.001). Interestingly, there was a strong positive correlation between the loss of a clinician's reputation and the loss of a client due to interpretive errors (*r* = 0.770, *p* < 0.001).

### 3.3 Gender and Professional Qualification Variations in the Perceptions of the Consequences of Interpretive Errors

Interestingly, female and male participants had different opinions about the likelihood of patient‐related and clinician‐related consequences of interpretive errors. More female participants (78%) considered undertreatment as likely patient‐related consequences of interpretive errors compared to male participants (63.6%). In contrast, more male participants considered patient harm or mortality (females, 40%; males, 30%) and negative impact on patient's quality of life (females, 40%; males, 28%) as the likely results of interpretive errors. When the clinician‐related consequences were compared, more female participants (84.8%) considered that legal consequences were more likely consequences compared to the male participants (77.4%). Female participants also considered loss of client (78.4% for females and 40.9% for males, *p* = 0.023) and loss of reputation (females, 82.5%; males, 54.6%; *p* = 0.039) were more likely to occur than male participants.

When the responses of general dental practitioners (GP) were compared with the specialists, it was found that more GPs (74.3%) considered that undertreatment was the most likely patient‐related outcome compared to specialist dentists (71.4%). More specialists (71%) thought that additional cost to the patient was a possible outcome compared to the GPs (60%). When the responses regarding the clinician‐related consequences were compared, trends were revealed. More specialists (71%) considered a hike in insurance premiums was likely to occur due to interpretive errors compared to GPs (54%). In contrast, more GPs (65.7%) considered loss of client as a more likely consequence of interpretive error compared to specialists (57%). For each patient‐related and clinician‐related consequence, dentists practising in public practice (hospital‐based) considered them more likely to occur than dentists in private practice.

### 3.4 Measures to Minimize Errors

**Figure 3:**
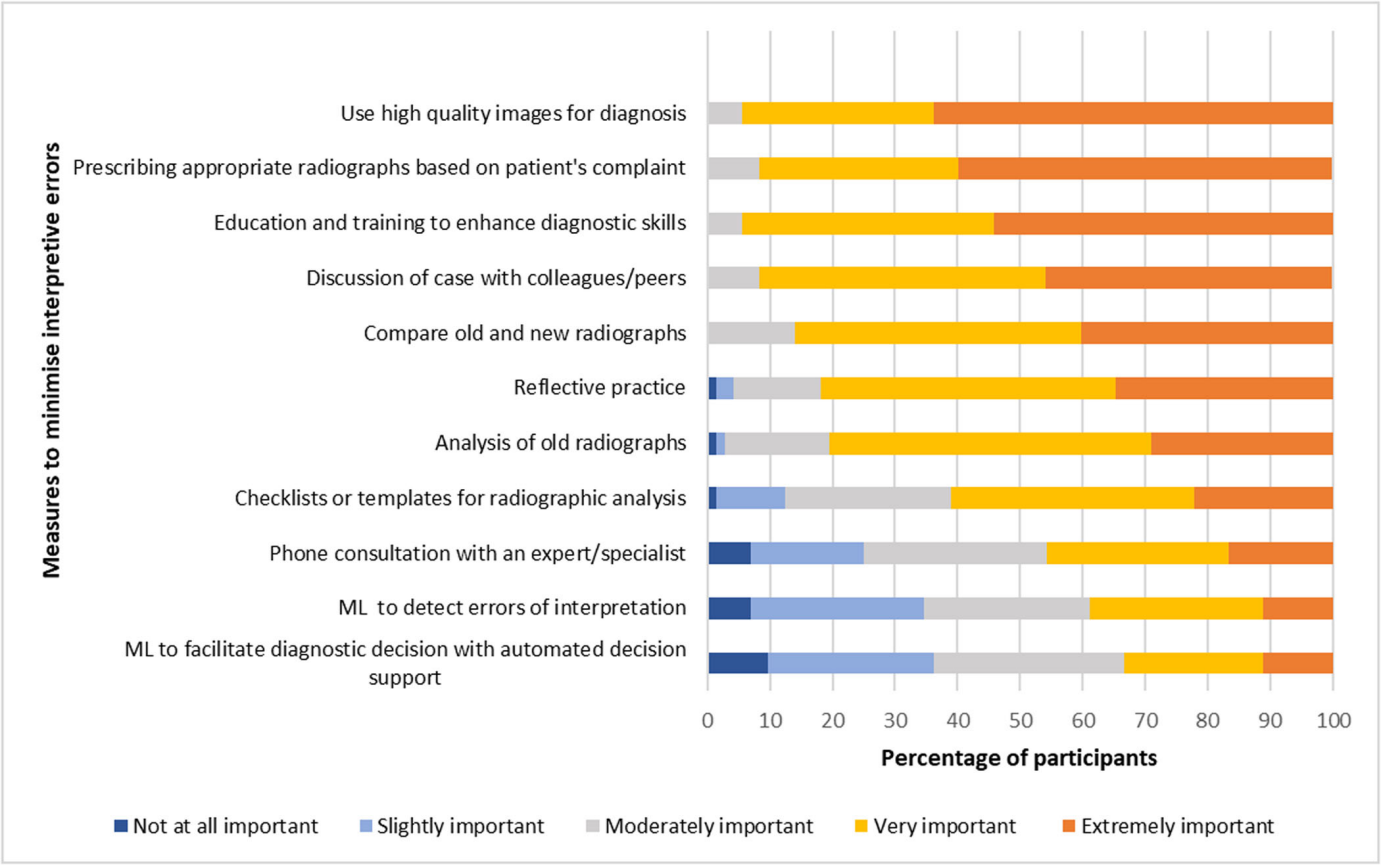
Measures to minimize interpretive errors.

Participants identified that using high‐quality images for diagnosis (63.9%) and prescribing appropriate radiographs based on the patient's chief complaint (59.7%) were the most effective means of minimizing interpretive errors. Undergoing training and education to enhance diagnostic skills (54.2%) and discussing a case with a colleague (45.8%) were also recognized as effective measures. Interestingly, although the use of ML algorithms was identified as the least effective measure (11.1%) for reducing the incidence of interpretive errors in dentistry (Figure 3), it was observed to correlate with measures related to cognitive aids such as standardized checklists (*p *< 0.001) (Table S2).

### 3.5 Gender Perspectives Regarding Measures to Minimize Interpretive Errors

Although there were no significant gender differences in the responses, more male participants (72.7%) considered using high‐quality radiographs as the essential measure to minimize interpretive errors compared to female participants (60.8%). More male participants (68%) considered prescribing appropriate radiographs a more effective error‐minimizing measure than females (58.6%). However, more female participants (43%) considered comparing new radiographs with old ones as an effective measure compared to males (36%). There were no differences in the responses between GPs and specialists and between dentists in public and private practices.

## 4 Discussion

The survey aimed to assess the dental practitioners' perceptions about the impact of interpretive errors in dentistry and potential solutions to minimize the occurrence of such errors. One notable observation was that the participants generally did not perceive interpretive errors led to serious harm to the patient. Instead, the survey revealed that dental practitioners placed greater emphasis on the potential consequences of interpretive errors in their practice, particularly in terms of the loss of reputation and financial consequences. This finding suggests that practitioners were more concerned about the impact of errors on their professional standing and the potential negative effects on their relationships with patients and colleagues.

The emphasis on loss of business and reputation as significant consequences of interpretive errors highlights the importance that dental practitioners place on maintaining a high level of competence and accuracy in their diagnostic and interpretive abilities. It underscores the potential impact that errors can have on patient trust and satisfaction and the potential for negative word‐of‐mouth and reputational damage. These findings contrast with medical radiology, where malpractice lawsuits are the biggest concern as a consequence of interpretive errors (Berlin 2017; Biswas et al. 2023). Other consequences of interpretive errors in medical radiology include repeat imaging, delayed or inappropriate treatment, avoidable hospital admissions, and increased mortality (Ahn et al. 2021; Onder et al. 2021). Overall, dental and medical practitioners share a common goal of minimizing interpretive errors. However, the concerns of dental practitioners are distinct from those of medical practitioners, which could be attributed to the unique contexts and priorities within each healthcare domain, such as variations in patient expectations, scope and impact of treatment provided, clinical practice dynamics, and legal considerations.

In countries like Australia, dental radiology diagnosis is mainly performed by general dentists. Admittedly, the findings of this study may not be generalizable to those countries with a higher number of DMFR specialists who provide radiological diagnosis and reporting. However, it is important to note that because general dentists perform routine dental images such as intraoral and panoramic radiographs and CBCT images, they are in a crucial position to detect jaw pathologies and malignancies at an early stage (MacDonald and Yu 2020; Price et al. 2012). Early detection of these conditions is essential to prevent disease progression, extensive destruction of dental supporting structures, and potential patient morbidity (Choi 2011). Therefore, targeted training and improving the diagnostic skills of general dentists can help minimize missed lesions, particularly because early‐stage diseases are often asymptomatic and may be identified incidentally on dental radiographs.

The survey also provided valuable insights into the interrelationships among the various consequences of interpretive errors. Participants considered that overtreatment negatively impacted their patients' quality of life and resulted in additional financial burdens on the clinician, legal consequences, and loss of the clinician's reputation. This highlighted the participant's awareness that negative patient outcomes were linked to financial and legal consequences for the clinicians.

The participants' perceptions of measures to minimize interpretive errors were also analyzed in this survey. The findings emphasize the significance of utilizing high‐quality images (taken using the correct technique and exposure settings and having optimal contrast, density, and brightness) and choosing appropriate radiographs based on patient symptoms. The impact of image quality has been investigated in medicine and dentistry (Boita et al. 2021; Bruno, Walker, and Abujudeh 2015; Ghazali, Mohd Yusof, and Norman 2021). These practices are crucial for improving diagnostic accuracy and reducing interpretive errors in dentistry. However, it is essential to consider that these higher quality images may not always align with the “As Low As Reasonably Achievable” (ALARA) principle, which prioritizes minimizing patient exposure. Further studies are needed to develop imaging protocols for specific dental conditions that balance the diagnostic efficacy with minimum radiation dose. Studies have shown that there is a greater likelihood of interpretive errors with 3D imaging compared to 2D imaging (Williams and Drew 2019). However, this survey did not study the impact that 2D versus 3D imaging may have on the incidence and consequences of interpretive errors.

Among the different measures to minimize interpretive errors, the participants also recognized training, education, and consulting with peers or colleagues as the most effective strategies. This emphasizes the importance of CPD and recognizes the value of collaboration and seeking input from others to enhance the accuracy of diagnosis and mitigate individual biases or oversights. Previous studies have shown that CPD can enhance clinical decision‐making skills and patient outcomes (Firmstone et al. 2013). The survey results could inform the design of training programs and implement quality assurance measures to minimize interpretive errors, improve patient care, and safeguard the professional reputation of dental clinicians.

Interestingly, the use of ML algorithms as cognitive aid was rated as the least effective measure for reducing the occurrence of interpretive errors, potentially due to scepticism about ML in dentistry. Studies have suggested that this scepticism may arise from concerns such as the interpretability and explainability of ML algorithms (Antoniadi et al. 2021). In addition, there are concerns that automated algorithms cannot fully replace or replicate the nuanced decision‐making capabilities of experienced dental professionals (Ding et al. 2023). There may also be concerns about patient privacy (Pethani 2021), liability, and responsibility for decisions made by the automated ML algorithms (Naik et al. 2022). It is important to note that this survey was conducted before the wider applications of ML algorithms and the development of large language models using generative AI. Given the advancements in technology since then, it is reasonable to consider that the perceptions of ML algorithms may have evolved if the survey were conducted in the present time.

The study's limitations include a small sample size, which may preclude the generalizability of the survey results. However, similar low participant numbers were noted in other Australian surveys of dental practitioners (Bulmer et al. 2022; George et al. 2017; Jadav and Abbott 2022). In addition, the low GP to dental specialist ratio may have limited the interpretation of the results in this survey, especially when comparisons were made between these two groups. Although the survey responses provided valuable insights, not all questions were answered by every participant. Future research will aim to validate the suggestions provided by the participants and explore the correlation between participant's perceptions about interpretive errors and their age and clinical experience. This correlation was not examined in this study due to the low sample size, which resulted in fewer participants in each age and clinical experience category. Furthermore, as this survey was voluntary, this may have resulted in selection bias.

## 5 Conclusions

The survey findings highlight the perceptions and beliefs of dental practitioners regarding the consequences of interpretive errors in dental radiology and potential solutions to minimize their occurrence. The results provide valuable insights to develop targeted interventions based on the clinician's level of training, gender, and sector of practice. Efforts to minimize interpretive errors should not only focus on improving patient safety but also address the concerns and priorities of dental practitioners, including the preservation of their professional reputation and business viability. However, there remains scepticism about the effectiveness of ML algorithms in dentistry. Future research is needed to explore the evolving perceptions of ML algorithms in dentistry and address these concerns effectively.

## Author Contributions

Shwetha Hegde has made substantial contributions to conceptualization, design, project administration, investigation and formal analysis, visualization, writing the original draft, and revising the manuscript. Jinlong Gao is involved in methodology, feedback on the concept, data analysis and writing (review and editing), and visualization. Stephen Cox participated in methodology, feedback on the concept, and writing (review and editing). Shanika Nanayakkara contributed to data curation and formal analysis, visualization, and writing (review and editing). Rajesh Vasa contributed to methodology, feedback on concepts, and writing (review and editing).

## Conflicts of Interest

The authors declare no conflicts of interest.

## Supporting information

Supporting information.

Supporting information.

Supporting information.

## Data Availability

The data that support the findings of this study are available from the corresponding author upon reasonable request.
