# Supplementary material for: Australian Dentists' Knowledge of the Consequences of Interpretive Errors in Dental Radiographs and Potential Mitigation Measures
Source: Clin Exp Dent Res. 2024 Oct 17;10(6):e70027. doi: 10.1002/cre2.70027 (PMC11486910; doi:10.1002/cre2.70027)
Supplement: Supplementary file 1 — Supporting information. [file CRE2-10-e70027-s001.docx]

Supplementary File 1: QUESTIONNAIRE

Dentist's Perceptions of Errors of Interpretation of Radiographs

**Introduction and Survey information**

**Summary of the research project:**

**Errors in the interpretation of medical imaging are the most common causes leading to diagnostic error. In clinical dentistry, the rate and cause of error in the interpretation of dental radiographs is not as well documented and studied. This survey aims to explore your perceptions of errors of interpretation on dental radiographs, the causes and implications of these errors and strategies to minimise them. The participant information statement attached here describes the study in further detail.**

**This survey will take approximately 20 minutes to complete.**

[**Participant Information Statement**](https://sydney.au1.qualtrics.com/CP/File.php?F=F_aXIvm15n9mye8ND)

**Thank you for taking the time to participate in the survey!**

**Section 1**

How important are **radiographs** in treatment planning for a patient?

Not at all important

Slightly important Moderately


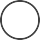
important

Very important Extremely


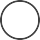
important


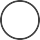

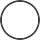

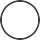


How often do you encounter errors of radiology interpretation in your practice?

When an error of radiology interpretation occurs, is it documented?


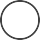

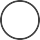
 Yes No

When an error in radiology interpretation occurs, is the patient informed?


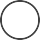
 Yes
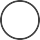
 No

To what extent do you feel that errors of radiology interpretation are preventable?

Never Sometimes About half the


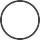

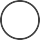
time


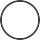


**Section 2: Frequency of errors**

Most of the time Always


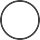

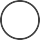


Please **rate in the order of likelihood**, type of error likely to occur in your practice?

1. **Error of Omission**: when no diagnosis is made
2. **Misdiagnosis**: when another diagnosis is made before the correct one
3. **Delayed Diagnosis**: when there is an unintentional delay in diagnosis
4. **Near Misses**: although an incident occurred that could have potentially lead to an adverse event if no harm came to the patient

Extremely unlikely


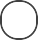


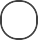


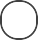


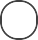


Somewhat unlikely


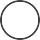


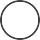


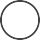


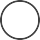


Neither likely nor unlikely


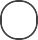


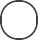


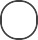


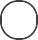


Somewha likely


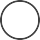


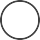


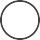


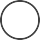


Please rank the **conditions** where errors of interpretation on radiographs are likely to occur;

dental caries assessment of depth

cracked tooth misdiagnosed as sinusitis/TMD/atypical facial pain misdiagnosis of late tooth development as hypodontia

failure to detect radiographic signs of periodontal disease failure to detect radiographic signs of an odontogenic cyst

failure to detect radiographic signs of benign odontogenic tumour cervical external root resorption misdiagnosed as root caries failure to diagnose internal root resorption

any other cause :

**Section 4: Contributing factors**

Please rate the following **potential causes** for errors of radiology interpretation:

1. When the dentist is not alert
2. When the dentist has an excessive workload
3. When treating the last patient of the day
4. When the dentist is unwell

Extremely unlikely


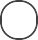

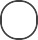

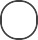

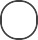


Somewhat unlikely


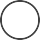

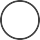

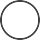

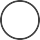


Neither likely nor unlikely


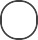

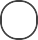

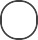

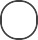


Somewha likely


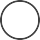

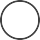

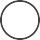

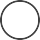


1.
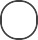

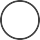

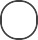

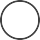
When the dentist is inexperienced- inadequate knowledge and skill
2. When a detailed history is not obtained
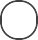

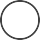

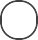

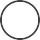

3. When treating a complex case
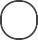

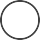

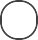

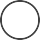

4. When treating a patient with dental fear
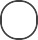

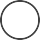

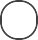

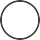

5. When treating a demanding patient
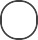

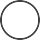

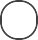

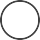

6. When treating a patient in pain
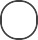

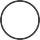

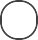

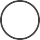

7.
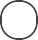

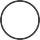

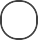

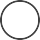
When there are distractions in the workplace

In your opinion, **how likely are the following to cause** errors of interpretation of radiographs ?

1. Reading a poor quality image
2. Mistaking a film fault for a pathology
3. Jumping to conclusions (not taking adequate time to analyse the radiograph)
4. Under-reading a radiograph( completely missing a lesion as a result)
5. Over reliance on previous interpretation of images
6. Over reliance on memory-may have seen a similar radiograph in the past
7. Failure to continue to analyse the image after an initial abnormality is detected
8. Completely missing lesions outside the area of interest

Extremely unlikely


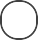

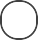

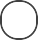


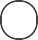


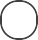


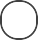


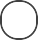


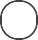


Somewhat unlikely


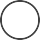

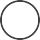

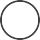


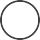


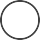


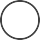


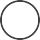


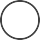


Neither likely nor unlikely


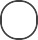

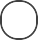

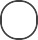


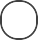


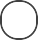


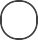


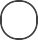


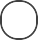


Somewha likely


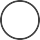

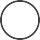


1. Misdiagnosis due to lesion mimicking another lesion
2. Underestimating the extent and severity of the lesion
3. Missing a diagnosis because of an inconspicuous lesion

**Section 5: costs and consequences of errors of interpretations**

In terms of **consequences of errors of interpretation to the patient**, please rate the likelihood of occurrence of following

1. Severe harm or mortality
2. Over treatment
3. Under treatment
4. Negatively affecting patient's quality of life-physical, mental
5. Additional financial cost to the patient

Extremely unlikely

Somewhat unlikely

Neither likely nor unlikely

Somewhat likely

Extremely likely

In terms of **consequences of errors of interpretation to the dentist**, please rate the likelihood of occurrence of following

Not at all important

Slightly important

Moderately important

Very important

Extremely important

1. Additional cost to the dentist to retreat the patient
2. Loss of business/client
3. Loss of reputation
4. Legal implications
5. Increase in the risk profile- insurance premiums

Not at all important

Slightly important

Moderately important

Very important

Extremely important

**SECTION 6: Strategies to reduce errors in interpretation of radiographs**

Please **rate the following activities in the order of their significance in reducing errors of interpretation** in the context of each patient

1. Seeking and analysing a patient's previous radiographs
2. Comparing a patient's current(latest) radiograph to their previous radiographs
3. Ensuring high quality images are used for diagnosis
4. Prescribing appropriate radiographs based on the patient's complaint

Not at all important

Slightly important

Moderately important

Very important

Extreme importa

Please **rate the significance** of the following in **reducing errors of interpretation** of dental radiographs

1. Using a checklist or a template for analysis of radiographic images to standardise the process
2. Having reflective practice to critically analyse clinical decisions
3. Phone consultation with an expert
4. Machine learning systems that detect errors of interpretation
5. Machine-learning systems to facilitate diagnostic feedback-automated decision support
6. Further education and training to enhance diagnostic skills
7. Discussion of cases with colleagues

**SECTION 7 : Participant Demographics**

Age

Gender

Not at all important

Slightly important

Moderately important

Very importa

Type of clinical practice : select all that is applicable

| general | public | metropolitan | none |
| --- | --- | --- | --- |
| specialist | private | rural |  |

other

Please indicate **number of hours spent per week** doing the following activities

clinical research teaching

administration

Please indicate the number of **years of clinical experience**?

Please indicate average number of your patients requiring radiographs per day?

Please select the types of radiographs frequently taken in your practice **and**

indicate the number of each radiograph taken per day

periapical radiographs bitewings

panoramic radiographs(OPG) other

Type of imaging system used for intraoral radiography in your practice

Photo stimulable direct digital chemically other

phosphor (PSP) (CCD/CMOS) processed/wet films

Phase 2 of this study involves focus group interviews to gain in depth understanding of the decision making processes involved in diagnoses on dental radiographs. It will also involve participation in a short radiographic diagnosis activity.

Would like to participate in the second phase of this study?

Yes No

If yes, please enter your email address

Would you like to receive future communication with the results of the study?

Yes No

If yes, please enter email address

[Powered by Qualtrics](http://www.qualtrics.com/)
