# Supplementary material for: Australian Dentists' Knowledge of the Consequences of Interpretive Errors in Dental Radiographs and Potential Mitigation Measures
Source: Clin Exp Dent Res. 2024 Oct 17;10(6):e70027. doi: 10.1002/cre2.70027 (PMC11486910; doi:10.1002/cre2.70027)
Supplement: Supplementary file 2 — Supporting information. [file CRE2-10-e70027-s003.docx]

Supplementary Table 1: Correlation coefficients for the consequences of interpretive errors

|  |  | Patient related consequences | | | | | Clinician related consequences | | | | |
| --- | --- | --- | --- | --- | --- | --- | --- | --- | --- | --- | --- |
|  | **Consequences of interpretive errors** | **Harm/mortality** | **Over treatment** | **Under treatment** | **Negative impact QOL** | **Additional cost to the patient** | **Additional cost to the dentist** | **Client loss** | **Reputation loss** | **Legal complications** | **Clinical experience** |
| Patient related consequences | **Harm/mortality** |  |  |  |  |  |  |  |  |  |  |
|  | **Over treatment** | 0.418** |  |  |  |  |  |  |  |  |  |
|  | **Under treatment** |  | 0.537** |  |  |  |  |  |  |  |  |
|  | **Negative impact QOL** | **0.605**** | 0.488** | 0.235* |  |  |  |  |  |  |  |
|  | **Additional cost to the patient** | 0.448** | 0.576** | 0.438** | 0.542** |  |  |  |  |  |  |
| Clinician related consequences | **Additional cost to the dentist** | 0.305** | 0.509** | 0.363** | 0.363** | 0.356** |  |  |  |  |  |
|  | **Client loss** |  |  | 0.231* |  |  | 0.427** |  |  |  |  |
|  | **Reputation loss** |  | 0.309** |  |  |  | 0.402** | **0.770**** |  |  |  |
|  | **Legal complications** |  | 0.231* | 0.243* |  |  | 0.309** | 0.453** | 0.549** |  |  |
|  | **Insurance impact**  Only statistically significant results are presented here.  *indicates p-value <0.05; **indicates p-value<0.001 |  |  |  |  |  | 0.414** | 0.388** | 0.498** | 0.399** | 0.256* |

Only statistically significant results are presented here.

*indicates p-value <0.05; **indicates p-value<0.001
