# Supplementary material for: Australian Dentists' Knowledge of the Consequences of Interpretive Errors in Dental Radiographs and Potential Mitigation Measures
Source: Clin Exp Dent Res. 2024 Oct 17;10(6):e70027. doi: 10.1002/cre2.70027 (PMC11486910; doi:10.1002/cre2.70027)
Supplement: Supplementary file 3 — Supporting information. [file CRE2-10-e70027-s002.docx]

|  | **Demographic data** | | **Measures to reduce interpretive errors** | | | | | | | | | | |
| --- | --- | --- | --- | --- | --- | --- | --- | --- | --- | --- | --- | --- | --- |
|  |  | **Age** | **Clinical Experience** | **Analysis of old radiographs** | **Compare old and new radiographs** | **High-quality images** | **Appropriate radiographs** | **Standardised checklist** | **Reflective practice** | **Expert consultations** | **ML for error detection** | **ML feedback** | **Further education** |
| **Measures to reduce interpretive errors** | **Compare old and new radiographs** |  |  | 0.561^**^ |  |  |  |  |  |  |  |  |  |
|  | **High quality images** | 0.307^*^ | 0.331^**^ | 0.392^**^ | 0.341^**^ |  |  |  |  |  |  |  |  |
|  | **Appropriate radiographs** | 0.310^*^ | 0.324^**^ | 0.316^**^ | 0.262^*^ | 0.648^**^ |  |  |  |  |  |  |  |
|  | **Standardised checklist** |  |  |  | 0.276^*^ | 0.333^**^ |  |  |  |  |  |  |  |
|  | **Expert consultations** | 0.267^*^ |  | 0.293^*^ |  | 0.350^**^ | 0.470^**^ | 0.374^**^ | 0.364^**^ |  |  |  |  |
|  | **ML for error detection** |  |  | 0.241^*^ | 0.258^*^ |  | 0.330^**^ | 0.514^**^ | 0.415^**^ | 0.462^**^ |  |  |  |
|  | **ML feedback** | 0.332^**^ |  | 0.280^*^ | 0.245^*^ |  | 0.235^*^ | 0.502^**^ | 0.293^*^ | 0.450^**^ | **0.838^**^** |  |  |
|  | **Further education** |  |  |  | 0.288^*^ | 0.446^**^ | 0.566^**^ | 0.289^*^ | 0.463^**^ | 0.341^**^ | 0.376^**^ | 0.303^**^ |  |
|  | **Discussion with colleagues** |  |  |  |  | 0.356^**^ | 0.390^**^ | 0.304^**^ | 0.477^**^ | 0.286^*^ | 0.265^*^ |  | 0.585^**^ |

Only statistically significant results are presented here.

*indicates p-value <0.05; **indicates p-value<0.001

Supplementary Table 2: Correlation coefficients for active measures and potential solutions to minimise the occurrence of interpretive errors.
